# Subcutaneous Ehrlich Ascites Carcinoma mice model for studying cancer-induced cardiomyopathy

**DOI:** 10.1038/s41598-018-23669-9

**Published:** 2018-04-04

**Authors:** Sneha Mishra, Ankit Kumar Tamta, Mohsen Sarikhani, Perumal Arumugam Desingu, Shruti M. Kizkekra, Anwit Shriniwas Pandit, Shweta Kumar, Danish Khan, Sathees C. Raghavan, Nagalingam R. Sundaresan

**Affiliations:** 1 Department of Microbiology and Cell Biology, Indian Institute of Science, Bengaluru, India; 2 Department of Biochemistry, Indian Institute of Science, Bengaluru, India

## Abstract

Cardiomyopathy is one of the characteristic features of cancer. In this study, we establish a suitable model to study breast cancer-induced cardiomyopathy in mice. We used Ehrlich Ascites Carcinoma cells to induce subcutaneous tumor in 129/SvJ mice and studied its effect on heart function. In Ehrlich Ascites Carcinoma bearing mice, we found significant reduction in left ventricle wall thickness, ejection fraction, and fractional shortening increase in left ventricle internal diameter. We found higher muscle atrophy, degeneration, fibrosis, expression of cell-adhesion molecules and cell death in tumor-bearing mice hearts. As observed in cancer patients, we found that mTOR, a key signalling molecule responsible for maintaining cell growth and autophagy was suppressed in this model. Tumor bearing mice hearts show increased expression and nuclear localization of TFEB and FoxO3a transcription factors, which are involved in the upregulation of muscle atrophy genes, lysosomal biogenesis genes and autophagy genes. We propose that Ehrlich Ascites Carcinoma induced tumor can be used as a model to identify potential therapeutic targets for the treatment of heart failure in patients suffering from cancer-induced cardiomyopathy. This model can also be used to test the adverse consequences of cancer chemotherapy in heart.

## Introduction

Cachexia is a common syndrome accompanying chronic aging related disorders, which increases the morbidity as well as mortality of the patients^1–3^. It is characterized by anorexia, loss of lean and fat body mass, dysregulation of metabolism, systemic inflammation and diverse cardiac pathologies^4,5^. Cardiac dysfunction is the manifestation of multifactorial diseases, such as chronic heart failure (CHF), cancer, renal failure, and metabolic disorders^1,6^. Cachexia is reported to be the cause for 20% of deaths in cancer patients, while 80% of all cancer patients suffer from it^4,7^. Cancer cachexia affects a wide variety of organs resulting in poor prognosis^4^. Most of the reports have focussed on skeletal muscle wasting in the context of cancer cachexia^2^. Nevertheless, dysregulation of cardiac functions has been found to be one of the major complications of cancer cachexia leading to heart failure. Cancer-induced cardiomyopathy results in cardiac dysfunction, similar to congestive heart diseases, which is accompanied by cardiac atrophy, metabolic remodelling, fibrosis and changes in the ultrastructure of the heart^8,9^. Similarly, cardiac atrophy is accompanied by interventricular and septal walls thinning and chamber dilation has been demonstrated in rodent models of cancer cachexia^10–14^.

Breast cancer is the second leading cause of deaths of women worldwide. Reports suggest that death in breast cancer patients are due to cardiovascular diseases^15^. In this study, we have tried to develop a model to study cardiovascular complications associated with breast cancer. We have used carcinogenic potential of the EAC cells to form subcutaneous tumours in 129/SvJ mice. Ehrlich Ascites Carcinoma (Ehrlich cells, EAC), a spontaneous murine mammary adenocarcinoma is a well-established model in tumor biology. EAC model has largely been used for study of tumor pathogenesis and development of anti-tumorigenic agents^16,17^. Previous studies have shown that subcutaneous EAC metastasizes to lungs, liver, spleen, kidney, bone, diaphragm, blood and adrenal glands^18^. To check the validity of our model, we tested the phenotype associated with cancer-cachexia. We have further studied changes in heart, which are commonly observed in cancer patients at both morphological and molecular levels along with its function.

Cachexia is characterized by an imbalance between protein synthesis and breakdown^9^, which is mediated by dysregulation of signal transduction pathways. Importantly, cardiac muscle atrophy accompanies downregulation of the mTOR signaling, which is major regulator of protein homeostasis^9^. Inactivation of mTORC1 has been shown to promote autophagy in cardiomyocytes^19^. In addition, the activity of TFEB, a mTOR-target transcription factor that specifically induces lysosomal biogenesis and autophagy is upregulated during muscle atrophy^20^. TFEB is phosphorylated by mTOR in nutrient rich conditions, resulting in its cytoplasmic localisation and inhibition of autophagy. In contrast to this phenomenon, starvation and mTOR inactivation result in increased nuclear localisation of TFEB, which leads to upregulation of the lysosomal-autophagic genes involved in muscle atrophy^21^. Studies indicate that the expression of atrophy inducing ubiquitin ligases Muscle Ring Finger-1 (MuRF-1) and Muscle Atrophy F-box (MAFbx-1/Atrogin-1) are increased in atrophic hearts^9^. Notably, FoxO family of transcription factors are major activators of MuRF-1 and Atrogin-1^9^.

Our study shows that subcutaneous EAC tumor induces cardiomyopathy in mice, which is associated with cardiac atrophy, fibrosis and dysfunction, a phenotype observed in human patients with cancer.

## Results

### Subcutaneous EAC tumor bearing mice develop cardiomyopathy

**Figure 1:**
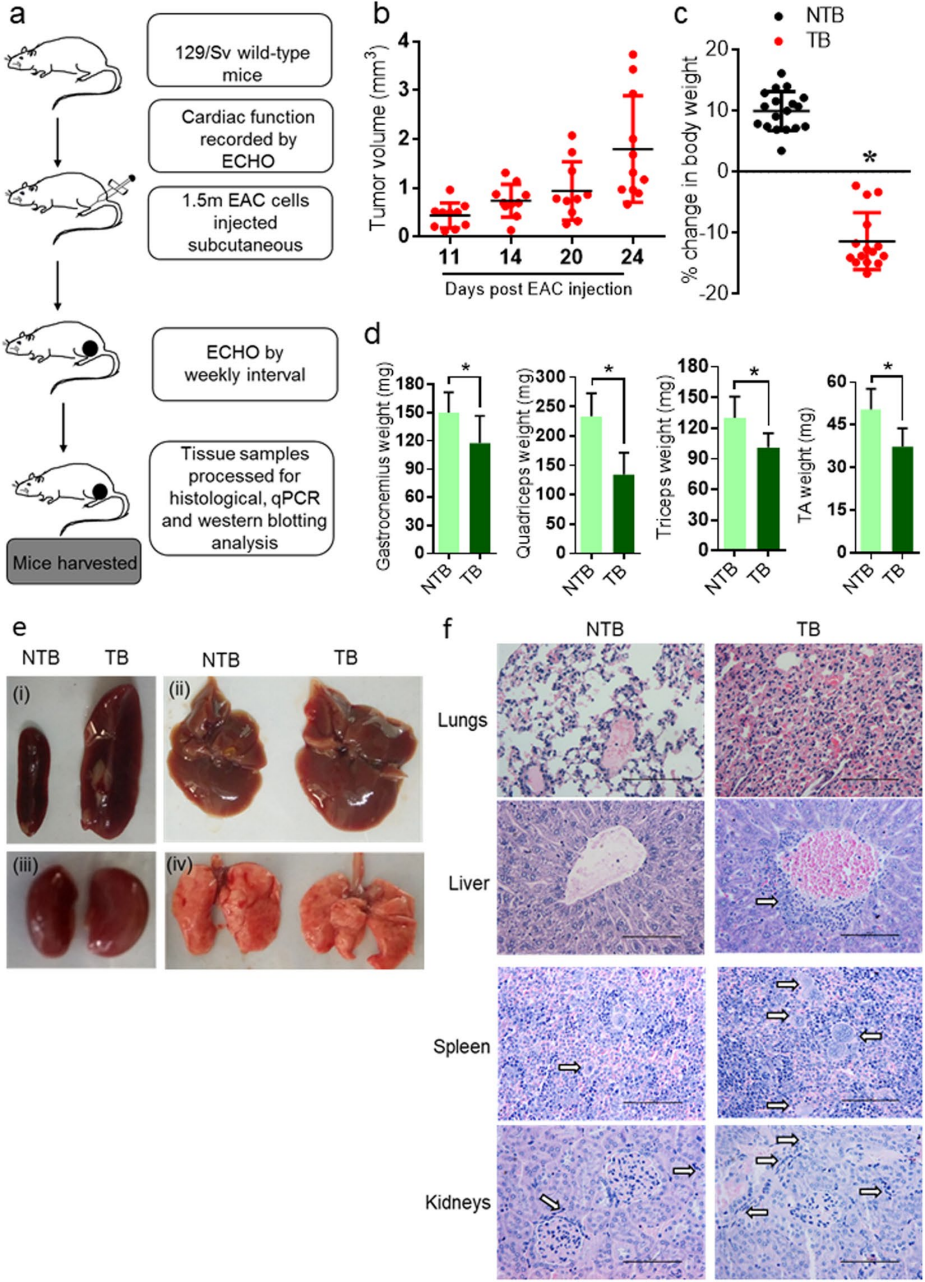
Subcutaneous EAC tumor induces cachexia in mice. (**a**) Schematic diagram of the model proposed to study breast cancer induced cachexia. (**b**) Graph representing tumor volume in mice. Tumor volume measured at different time points post EAC cells injection. n = 10–11 mice per group. (**c**) Graph representing percentage change in body weight of non-tumor bearing (NTB) and tumor bearing (TB) mice. n = 15–18 mice per group. (**d**) Graph depicting the weights of gastrocnemius, quadriceps, triceps and tibialis anterior (TA) muscle of non-tumor bearing (NTB) and tumor bearing (TB) mice. n = 6–10 mice per group. (**e**) Gross images of spleen (i), liver (ii), kidney (iii) and lungs (iv) of non-tumor bearing (NTB) and tumor bearing (TB) mice. n = 6 mice per group. (**f**) H&E staining images of lung, liver, spleen and kidney sections of NTB and TB mice demonstrating inflammatory cells infiltration (black arrows) in TB mice sections. n = 6 mice per group. Scale bar = 100 µm.

**Figure 2:**
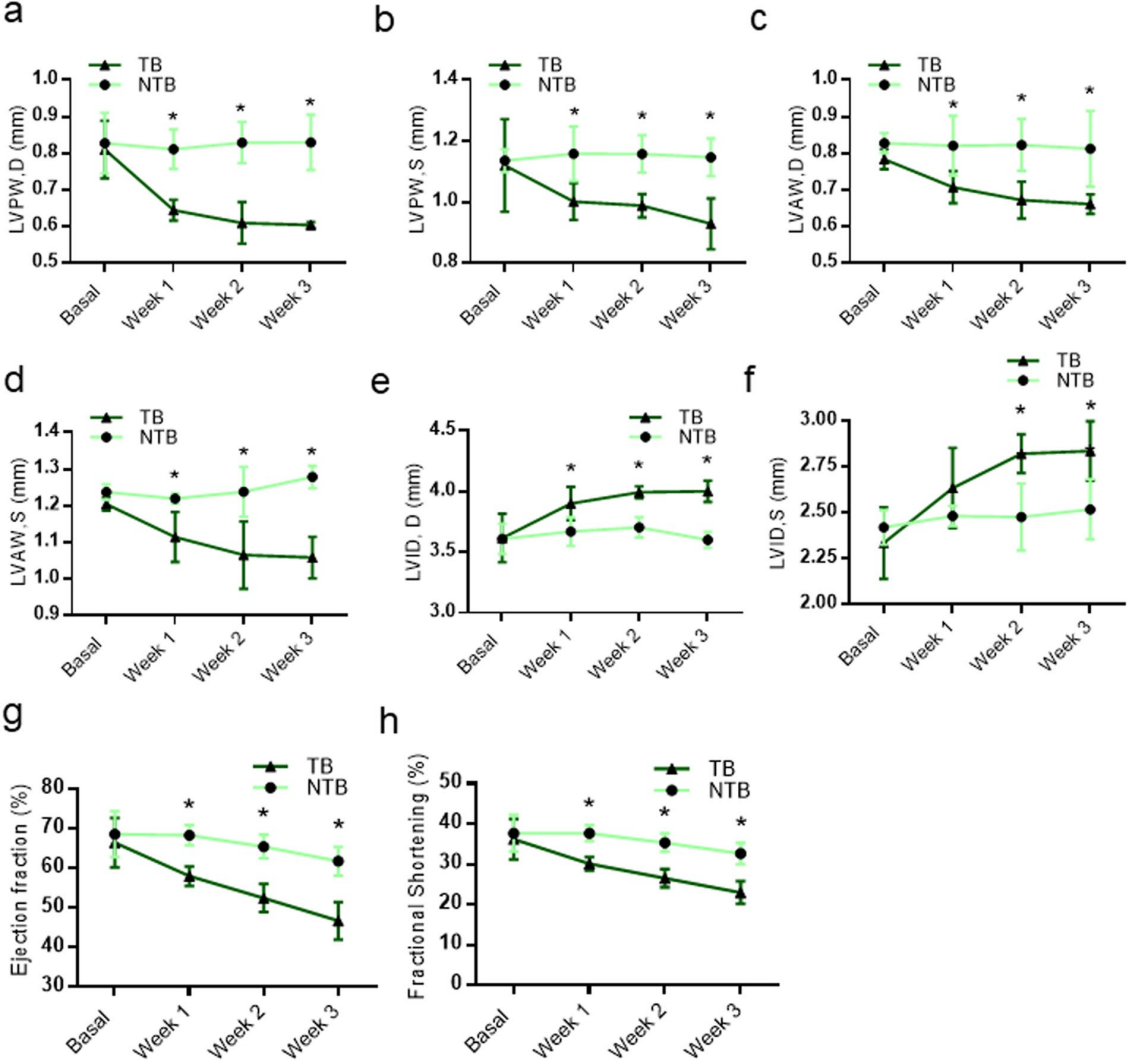
EAC subcutaneous tumor induces cardiac remodelling and contractile dysfunction in mice. (**a**) Graph showing posterior wall thickness at diastole of NTB and TB mice as assessed by echocardiography, n = 4–6 mice per group, Mean ± SD, *p < 0.05. (**b**) Graph showing posterior wall thickness at systole of NTB and TB mice as assessed by echocardiography, n = 3–6 mice per group, Mean ± SD, *p < 0.05. (**c**) Graph showing anterior wall thickness at diastole of NTB and TB mice as assessed by echocardiography, n = 4–6 mice per group, Mean ± SD, *p < 0.05. (**d**) Graph showing anterior wall thickness at systole of NTB and TB mice as assessed by echocardiography, n = 3–6 mice per group, Mean ± SD, *p < 0.05. (**e**) Graph showing left ventricular internal diameter at diastole of NTB and TB mice as assessed by echocardiography, n = 3–6 mice per group, Mean ± SD, *p < 0.05. (**f**) Graph showing left ventricular internal diameter at systole of NTB and TB mice as assessed by echocardiography, n = 3–6 mice per group, Mean ± SD, *p < 0.05. (**g**) Graph showing ejection fraction of NTB and TB mice as assessed by echocardiography, n = 5–6 mice per group, Mean ± SD, *p < 0.05. (**h**) Graph showing fractional shortening of NTB and TB mice as assessed by echocardiography, n = 5–6 mice per group, Mean ± SD, *p < 0.05.

Previous reports suggest that solid tumors induce cardiac dysfunction in cancer patients^22^. We were interested to test whether EAC tumors have similar effect on hearts of mice. We used Ehrlich Ascites Carcinoma cells to induce subcutaneous tumor in 129/SvJ mice and studied its effect on heart function. 129/SvJ mice were subcutaneously injected with 1.5 million EAC cells (Fig. 1a). We found concomitant reduction in body weight in TB mice with the progression of tumor which was not observed in NTB mice (Fig. 1b,c). Further, EAC tumor mice exhibited reduction in the weights of gastrocnemius, quadriceps, triceps and tibialis anterior (TA) muscles (Fig. 1d), a characteristic feature of cancer-cachexia. Previous studies suggest that EAC tumor metastases to various organs namely lung, liver, kidney, spleen and adrenal gland^18,23–25^. We tested whether in our model EAC tumor causes similar macroscopic and microscopic changes in various organs. Gross examination of various organs suggested that EAC tumor leads to enlargement of spleen, kidney, liver and lung (Fig. 1e), which are consistent with previous reports^25^. H&E staining showed degenerative changes and inflammatory cells infiltration to lungs, central vein in liver sections, cellular proliferation and increased megakaryocyte in the spleen, mild infiltration of inflammatory cells in the interstitial space of kidney (Fig. 1f), as reported in previous publication^24^. To analyse the effect of subcutaneous tumor on cardiac function, echocardiography was performed at weekly intervals after injection of EAC cells. Echocardiography data analysis suggests that tumor has significant effect on morphological features, such as wall thickness and left ventricular internal diameter (LVID) of TB mice hearts after a week of EAC cells injection. Left ventricular posterior and anterior wall thickness was significantly reduced in mice with EAC tumours (Fig. 2a–d). On contrast, LVID significantly increased in TB mice, when compared to NTB mice (Fig. 2e,f). Interestingly, cardiac functions, such as ejection fraction and fractional shortening started to deteriorate after 1 week of EAC cells injection (Fig. 2g,h). These data suggest that EAC tumor can affect both cardiac structure and function at an early stage, which is similar to cancer-induced cardiomyopathy as observed in humans.

### Subcutaneous Ehrlich ascites tumor induces atrophy, activation of fetal genes and fibrosis in heart

**Figure 3:**
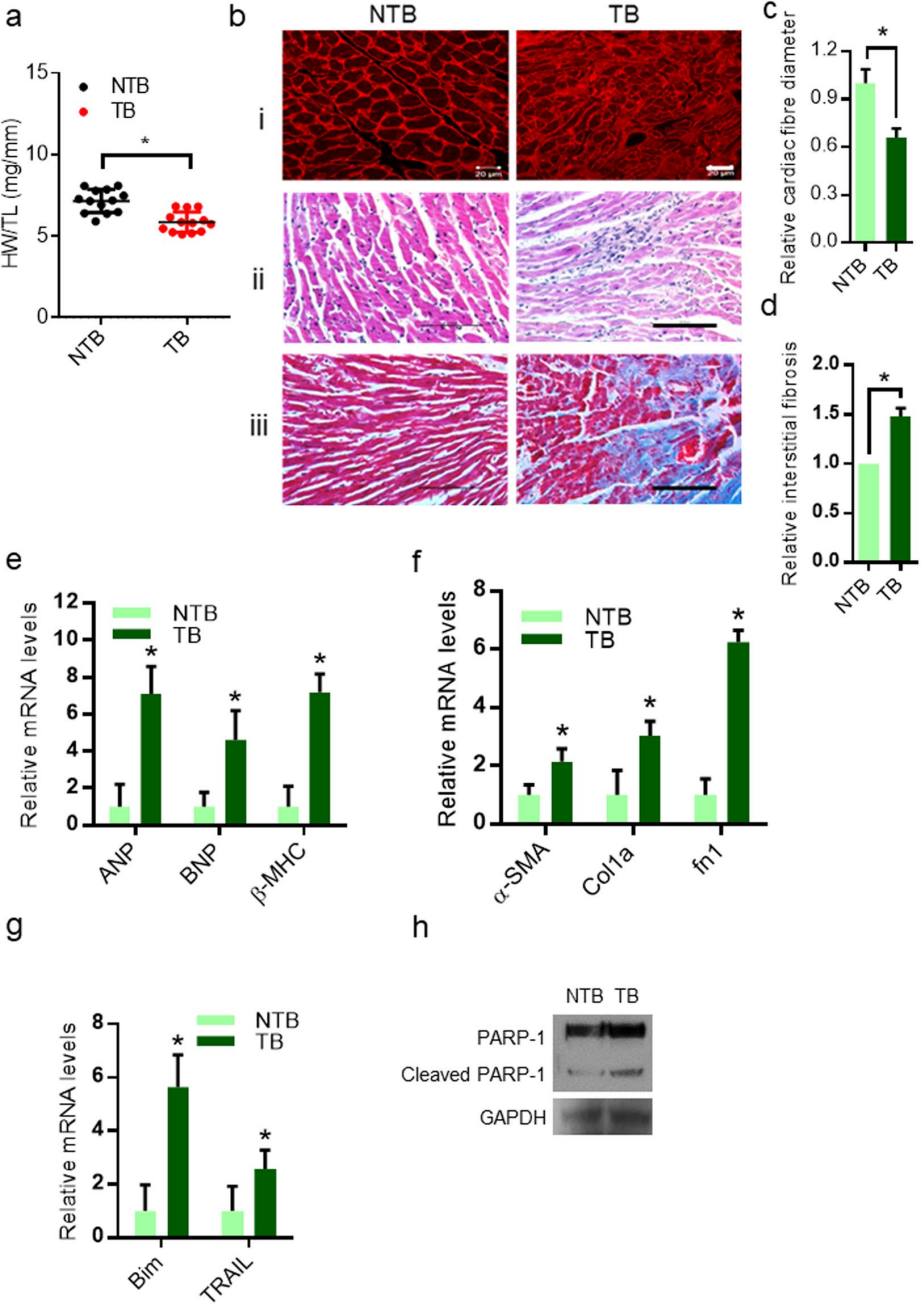
EAC subcutaneous tumor induces atrophy, fibrosis and degenerative changes in heart. (**a**) Graph showing Heart weight to tibia length ratio (HW/TL) of NTB and TB mice, n = 13 mice per group, Mean ± SD, *p < 0.05. (**b**–**i**) Confocal images representing WGA staining in heart sections of NTB and TB mice, Scale bar = 20 µm. (**b**–**ii**) Graph showing H&E staining in heart sections of NTB and TB mice showing degenerative changes including reduced muscle fibre diameter. (**b**-**iii**) Heart sections of NTB and TB mice stained with Masson’s trichrome stain showing cardiac fibrosis. (**c**) Graph showing relative fibre diameter in the cardiac sections of NTB and TB mice, quantified from Fig. 2(b-i). (**d**) Graph showing relative fibrosis in the cardiac sections of NTB and TB mice, quantified from Fig. 2(b-iii). (**e**) qPCR analysis of ANP, BNP and β-MHC in NTB and TB mice hearts, Mean ± SD, n = 4–5 mice per group, *p < 0.05. (**f**) qPCR analysis of α-SMA, Col1a and fn1 in NTB and TB mice hearts. Mean ± SD, n = 4–6 mice per group, *p < 0.05. (**g**) qPCR analysis of Bim and TRAIL in NTB and TB mice hearts, Mean ± SD, n = 5 mice per group, *p < 0.05. (**h**) Western blotting analysis of cleaved PARP-1 in NTB and TB mice heart samples.

Cardiac atrophy and degeneration of muscle fibres has been reported in cancer induced cardiomyopathy^9^. Therefore, we assessed the cardiac atrophy by measuring the heart weight/tibia length ratio. Results suggests that EAC tumor reduces heart weight by 14% in TB, when compared to NTB group (Fig. 3a). Measurement of the cross-sectional area of cardiac muscle fibres by staining with WGA suggests that the cardiac muscle fibre diameter, a marker of cardiac atrophy, was significantly reduced in TB mice, when compared to NTB mice hearts (Fig. 3b-i,c). Further, haematoxylin and eosin staining suggest that degenerative changes accompanied with reduced muscle fibre diameter in cardiac muscle sections of TB mice (Fig. 3b-ii). In the cancer patients, hearts with cardiomyopathy showed increased fibrosis^12^. To test whether the EAC TB mice showed fibrotic changes, we performed staining of collagen in cardiac tissue by Masson’s trichome stain. We found severe interstitial fibrosis in the cardiac tissue sections of TB mice (Fig. 3b-iii,d). Next, we checked the expression of fetal genes, such as Atrial natriuretic peptide (ANP), Brain natriuretic peptide (BNP), and β-myosin heavy chain (β-MHC), which are markers of cardiac remodeling^26^. Our results suggest fetal genes were upregulated in TB mice hearts (Fig. 3e). Next we tested whether the fibrotic changes occurs in TB mice hearts are due to generation of myofibroblasts^27^, we assessed the markers of myofibroblasts by qPCR analysis. We found that expression of myofibroblast markers such as alpha-smooth muscle actin (α-SMA), collagen, type I, alpha 1 (Col1a) and fibronectin 1 (Fn1) were significantly upregulated in TB mice hearts (Fig. 3f). These data indicate that subcutaneous EAC tumor induces cardiac fibrosis due to increased generation of myofibroblasts. Studies indicate that cancer-cachexia is associated with increased cell death^28–30^. Therefore, we assessed the expression of cell death markers by qPCR. The expression of BCL2-interacting mediator of cell death (Bim) and TNF-related apoptosis-inducing ligand (TRAIL), which are known to induce cell death in cardiomyocytes, were upregulated in the TB mice hearts (Fig. 3g). We further found that the levels of cleaved PARP-1, a marker of cell death, was high in the heart lysates of TB mice (Fig. 3h). These findings indicate that EAC tumor induces cellular and molecular changes in mice hearts, which mimic changes in cancer patient’s hearts. Collectively, our findings suggest that EAC-induced subcutaneous tumor induces cardiomyocyte atrophy, fibrosis and cell death in mice.

### Subcutaneous EAC tumor induces expression of lysosomal genes in mice heart

**Figure 4:**
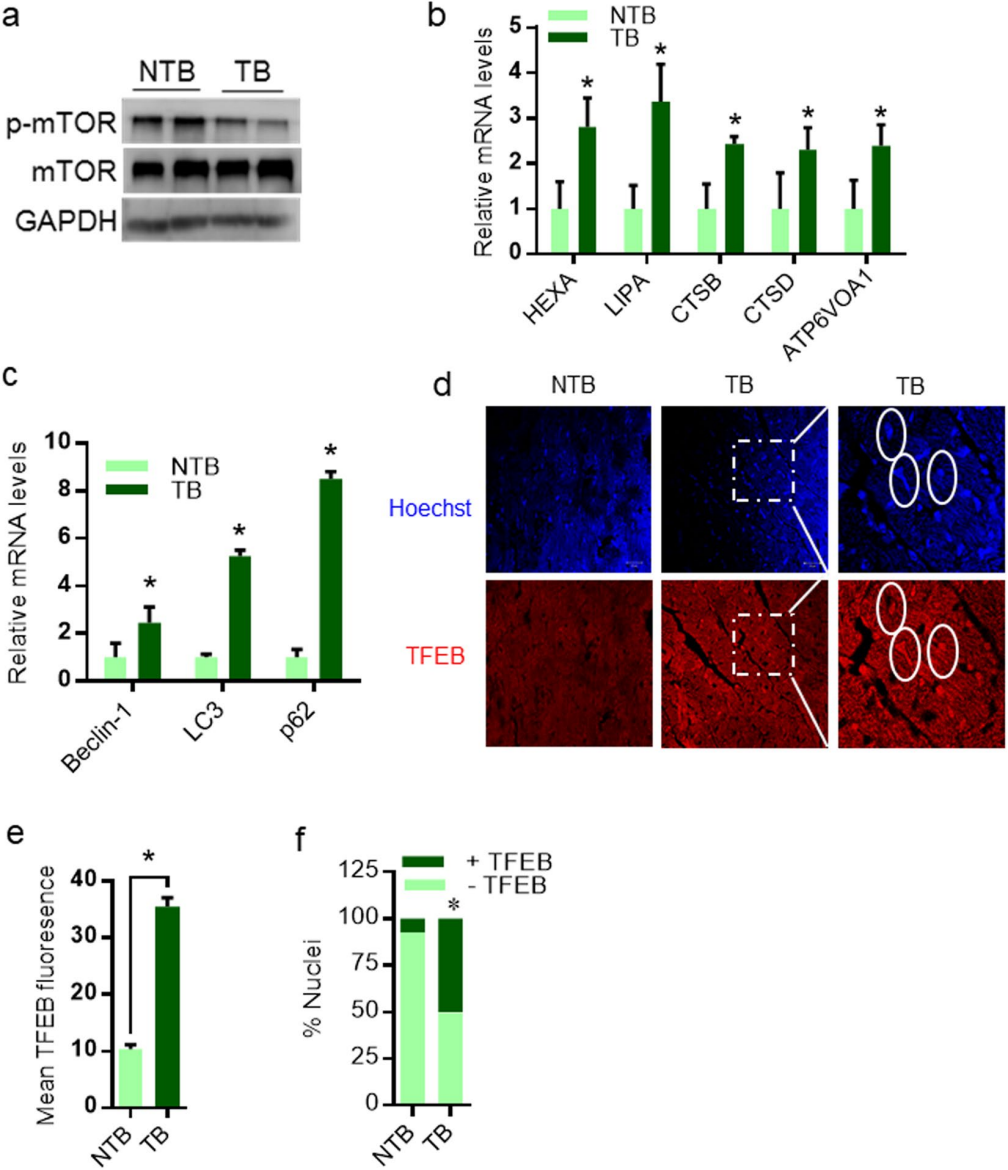
EAC subcutaneous tumor upregulates lysosomal genes in mice hearts. (**a**) Western blot analysis of NTB and TB mice hearts probed for p-mTOR and mTOR. GAPDH is used as loading control. n = 4 mice per group. (**b**) qPCR analysis of HEXA, LIPA, CTSB, CTSD and ATP6VOA1 in NTB and TB mice hearts, Mean ± SD, n = 4–5 mice per group, *p < 0.05. (**c**) qPCR analysis of Beclin-1, LC3 and p62 in NTB and TB mice hearts, Mean ± SD, n = 3–5 mice per group, *p < 0.05. (**d**) Representative confocal images of NTB and TB mice hearts stained for TFEB. Scale bar = 20 µm for left and middle images; n = 4 mice per group, Right side zoom-in images showing localisation of TFEB inside the nucleus in TB mice hearts marked by circles. (**e**) Graph showing quantification of TFEB levels in NTB and TB mice hearts. (**f**) Graph showing percentage of nuclei with and without TFEB localisation in NTB and TB mice hearts.

Previous reports suggest that mTOR signalling plays a critical role in inhibiting autophagy and lysosomal biogenesis in muscle to induce cachexia^31^. Our western blotting analysis showed phosphorylation of mTOR was reduced in TB mice hearts (Fig. 4a), suggesting that EAC tumor might promote autophagy and lysosomal biogenesis through suppression of mTOR signaling in heart. To assess the levels of lysosomal biogenesis genes and autophagy expression, we performed qPCR. We found that the expression of lysosomal biogenesis genes such as hexosaminidase A (HEXA), lipase A (LIPA), cathepsin B (CTSB), cathepsin D (CTSD) and ATPase, H^+^ transporting, lysosomal V0 subunit A1 (ATP6VOA1) was significantly upregulated in hearts of TB mice (Fig. 4b). Similarly, the expression of Beclin-1, LC3 and p62, which are well studied autophagy genes were also upregulated, indicating that EAC tumor induces lysosomal biogenesis and autophagy, which could be causing atrophy in mice hearts (Fig. 4c). Previous studies indicate that mTOR signalling inhibits lysosomal biogenesis and autophagy by negatively regulating the transcription factor TFEB^21^. Since, we found reduced mTOR phosphorylation in heart samples of EAC TB mice, we tested the expression and nuclear localization of TFEB by confocal microscopy. Our results indicate that TB mice hearts show significantly higher expression as well as nuclear localization of TFEB (Fig. 4d–f). These data suggest that EAC tumor promotes autophagy and lysosomal biogenesis through suppression of mTOR.

### Subcutaneous EAC tumor induces expression of atrophy-related genes in mice heart

**Figure 5:**
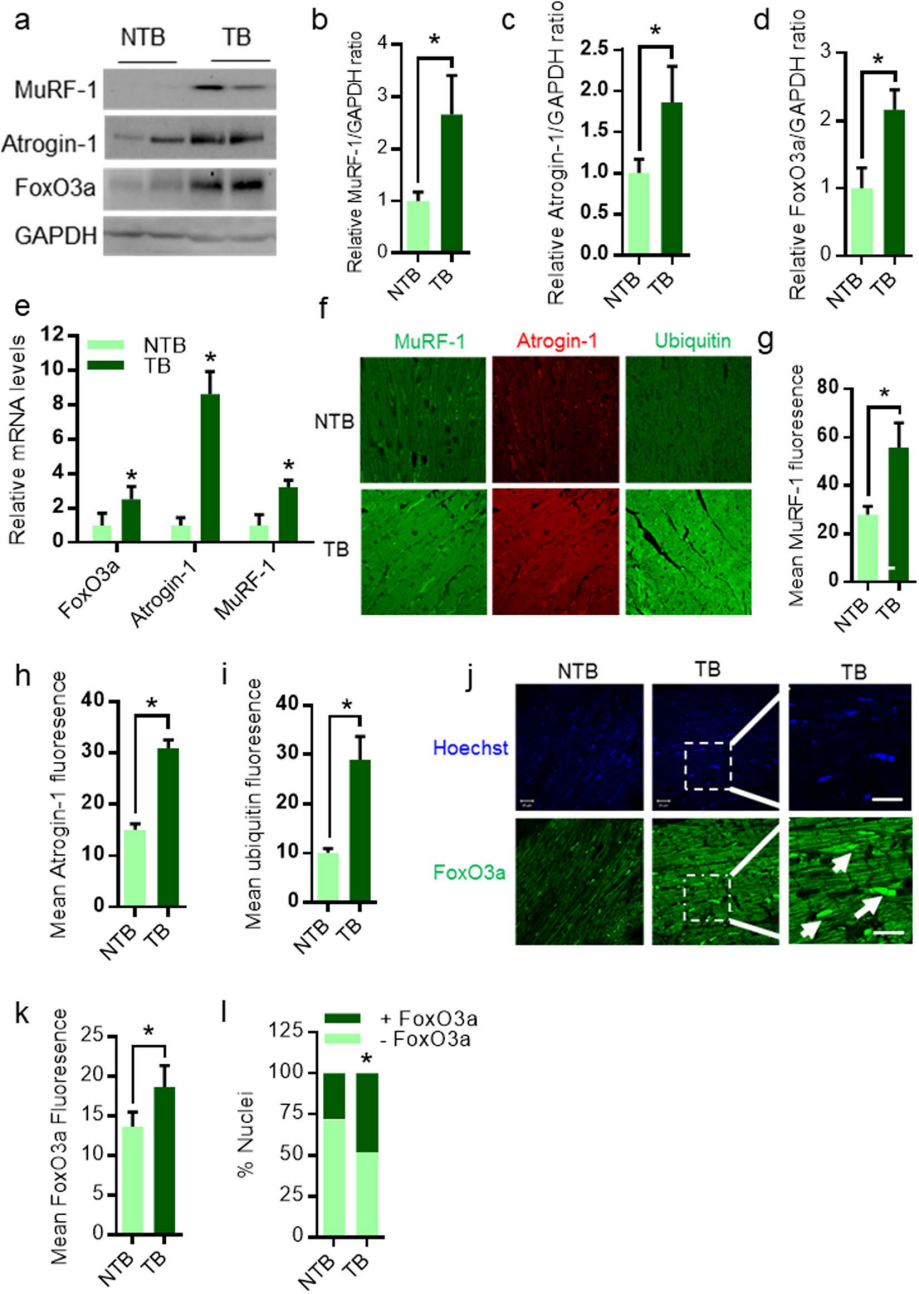
EAC subcutaneous tumor upregulates atrophy genes in mice hearts. (**a**) Western blot analysis of NTB and TB mice hearts probed for MuRF-1, Atrogin-1 and FoxO3a. GAPDH is used as loading control. n = 7–8 mice per group. (**b**) Graph representing quantification of MuRF-1/GAPDH levels in NTB and TB mice hearts. n = 7–8 mice per group. (**c**) Graph representing quantification of Atrogin-1/GAPDH levels in NTB and TB mice hearts. n = 4–5 mice per group. (**d**) Graph representing quantification of FoxO3a/GAPDH levels in NTB and TB mice hearts. n = 3 mice per group. (**e**) qPCR analysis of FoxO3a, Atrogin-1 and MuRF-1 in NTB and TB mice hearts. Mean ± SD, n = 5–7 mice per group, *p < 0.05. (**f**) Representative confocal images of NTB and TB mice hearts stained for MuRF-1, Atrogin-1 and total-ubiquitin levels, Scale bar = 20 µm. (**g**) Graph showing quantification of MuRF-1 in NTB and TB mice hearts. (**h**) Graph showing quantification of Atrogin-1 in NTB and TB mice hearts. (**i**) Graph showing quantification of total cellular ubiquitin levels in NTB and TB mice hearts. (**j**) Representative confocal images of NTB and TB mice hearts stained for FoxO3a, Scale bar = 20 µm for left and middle images; n = 4 mice per group, Right side zoom-in images showing localisation of FoxO3a inside the nucleus in TB mice hearts marked by white arrows. (**k**) Graph showing quantification of FoxO3a expression levels in NTB and TB mice hearts. (**l**) Graph showing percentage of nuclei with and without FoxO3a localisation in NTB and TB mice hearts.

Studies indicate that the transcription factor FoxO3a regulate the atrophy program by regulating the expression of ubiquitin-ligases such as atrogin-1 and MuRF-1 in heart^9^. Since previous reports suggest that upregulation of atrogin-1 and MuRF-1 are responsible for the muscle degeneration in cancer patients^9^, we tested the expression levels of atrogin-1, MuRF-1 and FoxO3a by qPCR analysis and western blotting. Our results indicate that EAC tumor promotes expression of atrogin-1, MuRF-1 and FoxO3a at both mRNA and protein levels in TB mice hearts (Fig. 5a–e). To further validate our findings, we performed IHC in the heart tissue sections of TB mice. We found that EAC tumor significantly increased the expression of atrogin-1, MuRF-1 and total cellular ubiquitin levels, suggesting that EAC tumor induces atrophy program in heart (Fig. 5f–i). Next, we tested the expression levels and nuclear localization of FoxO3a in heart tissues of TB mice. Interestingly, the heart samples of TB mice showed increased FoxO3a levels (Fig. 5j,k). Further confocal microscopy image analysis suggested that TB mice heart sections show markedly higher nuclear FoxO3a levels (Fig. 5j,l), suggesting that EAC tumor induces FoxO3a transcription factor, which might be one of the major cause for cardiac atrophy in TB mice.

## Discussion

Cancer induced cardiac remodelling involves changes in wall thickness, ventricle diameter, and contractile dysfunction^9^. Fetal gene reprogramming also occurs in the hearts of cancer-induced cardiomyopathy^32,33^. In our EAC model, we found marked atrophy, fibrosis and contractile dysfunction in hearts of mice bearing tumor. Our mice model can be used to identify novel therapeutic candidates, explore novel signalling pathways and test drugs for treating cancer-induced cardiomyopathy. This is the first publication describing a method to test how breast cancer affects cardiac structure and function using a novel mice model.

EAC cells are originally derived from murine mammary tissue, and they are undifferentiated, and hyper-diploid in nature^34,35^. EAC cells are aggressive with 100% tumorigenic ability^34^. Previous reports suggest metastatic behaviour of EAC in lungs, liver, spleen, kidney, bone, diaphragm, blood and adrenal glands^18^. EAC form free neoplastic cells in peritoneal ascites fluid of mice and can be passaged and maintained in peritoneal cavity of the mice and rats^36^. Mice used for passaging the EAC cells survive up to 22 days^37^. With increase in the number of passages, the aggressiveness of EAC cells increases. A major advantage of using ascites carcinoma cells is exact number of cells injected in mice can be monitored. Since, neoplastic cells grow as free suspension, no mechanical stress is needed for the disintegration of cells.

Most of the studies on cancer cachexia have been performed in Colon-26 (C26) adenocarcinoma model of cancer cachexia^38^. Previously studies conducted in C26 and ApcMin/ + mice model have shown that solid tumor induces cardiac dysfunction. Our work demonstrates similar findings and propose that cancer induced cardiomyopathy may be a universal phenomenon in cancer patients. A portion of cancer patients die due to cardiovascular complications as the treatment regimen including anticancer drugs, such as anthracycline and fluorouracil. These drugs can have side-effects on the heart function, in addition to cancer induced cardiac malfunctions^39,40^. Though, we have not tested the anticancer agents in our model, we believe that EAC model of cancer-induced cardiomyopathy can be used for assessing the simultaneous effects of anticancer agents on tumor and the heart.

In rodent models of cancer cachexia, atrophy of the left ventricle accompanied by thinning of the interventricular and septal walls and chamber dilation has been demonstrated^10–14^. However, the cardiovascular complications arising from the cancer itself varies considerably between human patients. Interestingly, gastrointestinal, pancreatic, and non-small cell lung cancer patients with cardiac cachexia exhibit reduced heart mass and left ventricle remodelling, including LV wall thinning and increased fibrosis^9^. There are several lines of evidences suggest that reduced ventricle volume, but not chamber dilation is common in human patients with cancer cachexia^41,42^, which is quite opposite phenomenon to dilation observed in animals. Therefore, our model may not recapitulate the cardiac remodelling observed in human cancer cachexia, although it mirrors the molecular changes found in human cancer cachexia.

Increase in protein degradation via ubiquitin-proteasome pathway as well as autophagy is one of the major causes of cardiomyopathy associated with cancer-cachexia^11,13^. Studies indicate that the suppression of the mTOR signalling is the major cause for cardiomyopathy associated with cancer-cachexia^31^. Our results of reduced phosphorylation of mTOR in TB mice hearts, are consistent with previous reports of reduced mTOR activity in degenerating muscle^31^. Many differentially expressed genes have been found in both cardiac and skeletal tissue in C26 model of cancer-cachexia^43^. Multiple lines of evidence suggest that increased expression of muscle specific E3-ubiquitin ligases, and lysosomal genes are causes of cachexia^44^. In our work, we found both arms of muscle degeneration, i.e. autophagy/lysosome and ubiquitin mediated degradation, are upregulated in heart of EAC model of cancer-cachexia. It is possible that the EAC tumor might suppress the activity of mTOR in heart, which could contribute to the hyperactivation of TFEB and FoxO3a, resulting in increased proteolysis by autophagy and proteasome degradation. Overall, these molecular events might induce cardiac muscle degeneration in EAC tumor bearing mice.

The data presented herein demonstrate, for the first time, development of a novel model system to study the breast cancer-induced cardiomyopathy. Our model can be used to identify potential therapeutic targets for the treatment of cancer induced cachexia. Similarly, this model can be used to test the effect of various drugs used in chemotherapy on heart function. Interestingly, our cancer induced cachexia model can be used in diverse genetic backgrounds and in genetically modified mice to understand the role of a specific protein(s) in the pathogenesis of cancer-induced cardiomyopathy.

## Materials and Methods

### Animals

All animal experiments were carried out with the approval of the Institutional animal ethics committee of Indian Institute of Science, Bengaluru, India constituted as per the article number 13 of the Committee for the Purpose of Control and Supervision of Experiments on Animals (CPCSEA), Government of India. We performed all animal experiments in accordance with CPCSEA guidelines for animal handling and welfare. Both tumor bearing (TB) and non-tumor bearing (NTB) 129/SvJ mice were housed in Central Animal Facility, Indian Institute of Science. Chow diet and water were given *ad libitum*. Male 129/SvJ mice were kept in 12/12 hours light-dark cycle. At 2 months of age, half of the group at random were inoculated subcutaneously in the right flank with 1.5 million EAC cells while the other half were injected with PBS. Mice were monitored carefully, and the size of the tumor was measured by Vernier calliper. Tumor volume was measured using standard formula, *L* × *W* ^2^ × 0.52, where *L* denotes largest diameter and *W* denotes shortest diameter.

### Maintenance of EAC cells

EAC cells were maintained in male BALB/c mice. EAC cells were passaged as per the standard protocols^45^. Briefly 1 ml of sterile PBS was injected in peritoneal cavity of BALB/c mice to dilute the liquid tumor. Diluted EAC cells were withdrawn from these mice, and 200 µl of the cells were injected intraperitoneally in 4–6 weeks old male BALB/c mice and maintained for 1 week till next passage.

### Echocardiography

VisualSonics Vevo 1100 machine was used for performing echocardiography. Mice were anesthetized by isoflurane (1%) inhalation, and chest hair were removed using a topical hair removal agent.

### Histology

NTB and TB mice were harvested after 24 days of injection and heart tissues were fixed immediately in neutral buffered formalin (10%) and preserved until processed further. Heart tissue was processed using automated tissue processor (Leica, Germany). H&E staining was performed to evaluate the muscle fibre diameter in the cardiac tissues. Fibrosis in heart tissues was visualized using Masson’s trichome (Sigma) staining of paraffin embedded 4 µm thick tissue sections. Immunohistochemistry was used to visualize muscle fibre diameter after staining with wheat germ agglutinin (Molecular probes).

### Western blot analysis

Previously reported protocol was used for western blotting^46^. Mouse heart tissue lysate was prepared for western blot with lysis buffer containing 50 mM Tris-HCl, 150 mM NaCl, 1 mM EDTA, 1 mM EGTA, pH 7.8, and 1% Triton X100 supplemented with sodium ortho-vandate, sodium pyrophosphate, protease inhibitor cocktail (Sigma) and PMSF. Antibodies used are as follows: mTOR (Millipore), p-mTOR (Santa Cruz), ubiquitin (Millipore), Atrogin-1 (Thermo Scientific), MuRF-1 (Santa Cruz), FoxO3a (Millipore) and GAPDH (Santa Cruz).

### Confocal microscopy

Immunofluorescence protocol has been described in our previous publication^47^. Fixed tissue sections were incubated with the primary antibodies, followed by incubation with secondary antibodies conjugated with fluorophore. Hoechst was used to stain the nuclei. Carl-Zeiss LSM 710 confocal microscope was used to acquire images.

### Real-time qPCR analysis

Protocol for real-time qPCR has been described in our previous publication^46^. The primer sequences used are ANP For.: CCTGTGTACAGTGCGGTGTC, ANP Rev.: CCTCATCTTCTACCGGCATC; BNP For.: AAGGGAGAATACGGCATCATTG, BNP Rev.: ACAGCACCTTCAGGAGATCCA; β-MHC For.: AGCAGCAGTTGGATGAGCGACT, β-MHC Rev.: CCAGCTCCTCGATGCGTGCC; α-SMA For.: CTGACAGAGGCACCACTGAA, α-SMA Rev.: CATCTCCAGAGTCCAGCACA; Col1a For.: TGCTGCTTGCAGTAACGTCG, Col1a Rev.: TCAACACCATCTCTGCCTCG; Fn1 For.: GCGGTTGTCTGACGCTGGCT, Fn1 Rev.: TGGGTTCAGCAGCCCCAGGT; Bim For.: CGACAGTCTCAGGAGGAACC, Bim Rev.: CCTTCTCCATACCAGACGGA; TRAIL For.: CGGAGAAGCAACTCAGCTTTA, TRAIL Rev.: GTTGAGAAATGAATGCCCTTTCC; FoxO3a For.: AAACGGCTCACTTTGTCCCA, FoxO3a Rev.: TTGTGCCGGATGGAGTTCTTC, Atrogin-1 For.: AGCGCTTCTTGGATGAGAAA, Atrogin-1 Rev.: GGCAGTCGAGAAGTCCAGTC; MuRF-1 For.: TGCCTGGAGATGTTTACCAAGC, MuRF-1 Rev.: AAACGACCTCCAGACATGGACA; HEXA For.: ACCTGGGAGGGGATGAAG, HEXA Rev.: ATGAAGGCCTGGATGTTGG; LIPA For.: CTAGAATCTGCCAGCAAGCC, LIPA Rev.: AGTATTCACCGAATCCCTCG; CTSB For.: TTTGATGCACGGGAACAATG, CTSB Rev.: TTGGTGTGAATGCAGGTTGC; CTSD For. CCGGCGTCTTGCTGCTCA, CTSD Rev.: TTGCGCAGAGGGATTCTGAT; ATP6VOA1 For.: ACCGTGGCTATCCTGCTG, ATP6VOA1 Rev.: CCCAGTGTAGAATTTGTTCTGGA; Beclin-1 For.: AATCTAAGGAGTTGCCGTTATAC, Beclin-1 Rev.: CCAGTGTCTTCAATCTTGCC; LC3 For.: CGT CCT GGA CAA GAC CAA GT, LC3 Rev.: ATTGCTGTCCCGAATGTCTC; p62 For.: CGCCTTCATCCGAGAAAC, p62 Rev.: GCTGCCCTATACCCACATCT; Actin For.: TTCTACAATGAGCTGCGTGTG, Actin Rev.: GGGGTGTTGAAGGTCTCAAA; GAPDH For.: TATGTCGTGGAGTCTACTGGT, GAPDH Rev.: GAGTTGTCATATTTCTCGTGG.

### Quantification and statistical analysis

Graph-pad prism version 6.04 was used for data analysis and plotting graphs. Data analysis was performed by t-test, one-way ANOVA, and two-way ANOVA, where * signifies p value less than 0.05. Confocal images were analysed using ZEN and ImageJ. Densitometric analysis was performed by ImageJ.

### Data Availability

The data generated during and/or analysed during the current study are available from the corresponding author on reasonable request.

## Electronic supplementary material

Supplementary Information
