# Supplementary material for: Subcutaneous Ehrlich Ascites Carcinoma mice model for studying cancer-induced cardiomyopathy
Source: Sci Rep. 2018 Apr 4;8:5599. doi: 10.1038/s41598-018-23669-9 (PMC5884778; doi:10.1038/s41598-018-23669-9)

Subcutaneous Ehrlich Ascites Carcinoma mice model for studying cancer-induced cardiomyopathy

Sneha Mishra[1], Ankit Kumar Tamta[1], Mohsen Sarikhani[1], Perumal Arumugam Desingu[1], Shruti M. Kizkekra[1], Pandit Anwit Shriniwas[1], Shweta Kumar[1], Danish Khan[1], Sathees C. Raghavan[2], Nagalingam R. Sundaresan[1,#]

Figure 3h

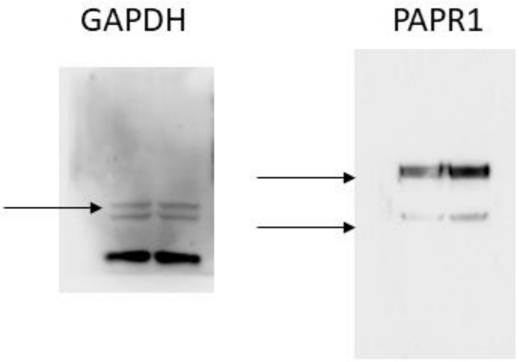

Figure 4a

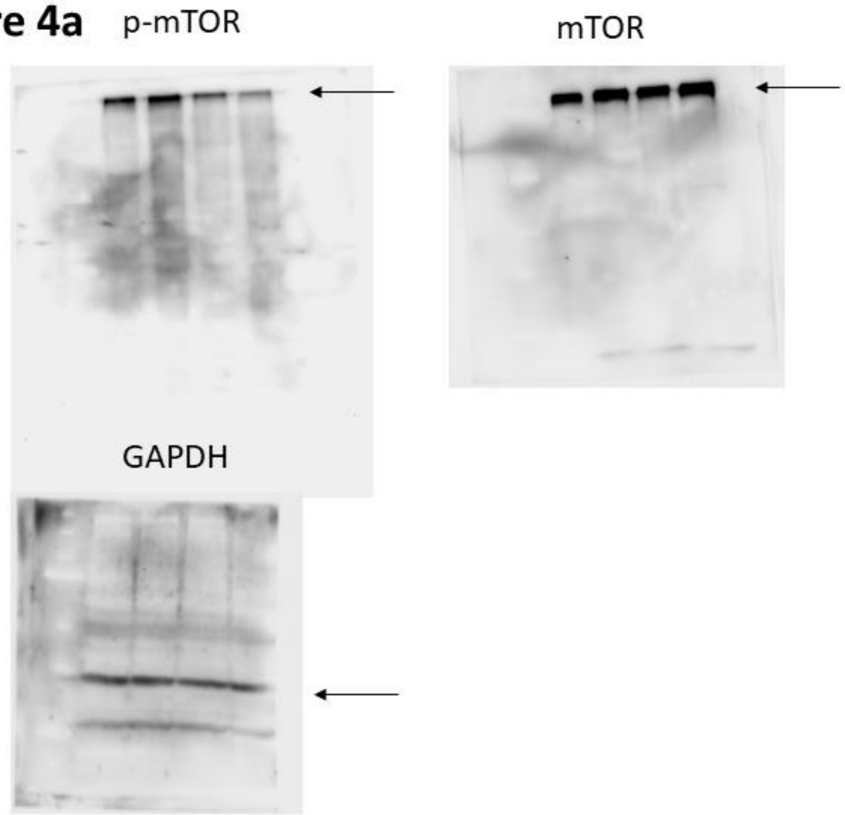

Figure 5a

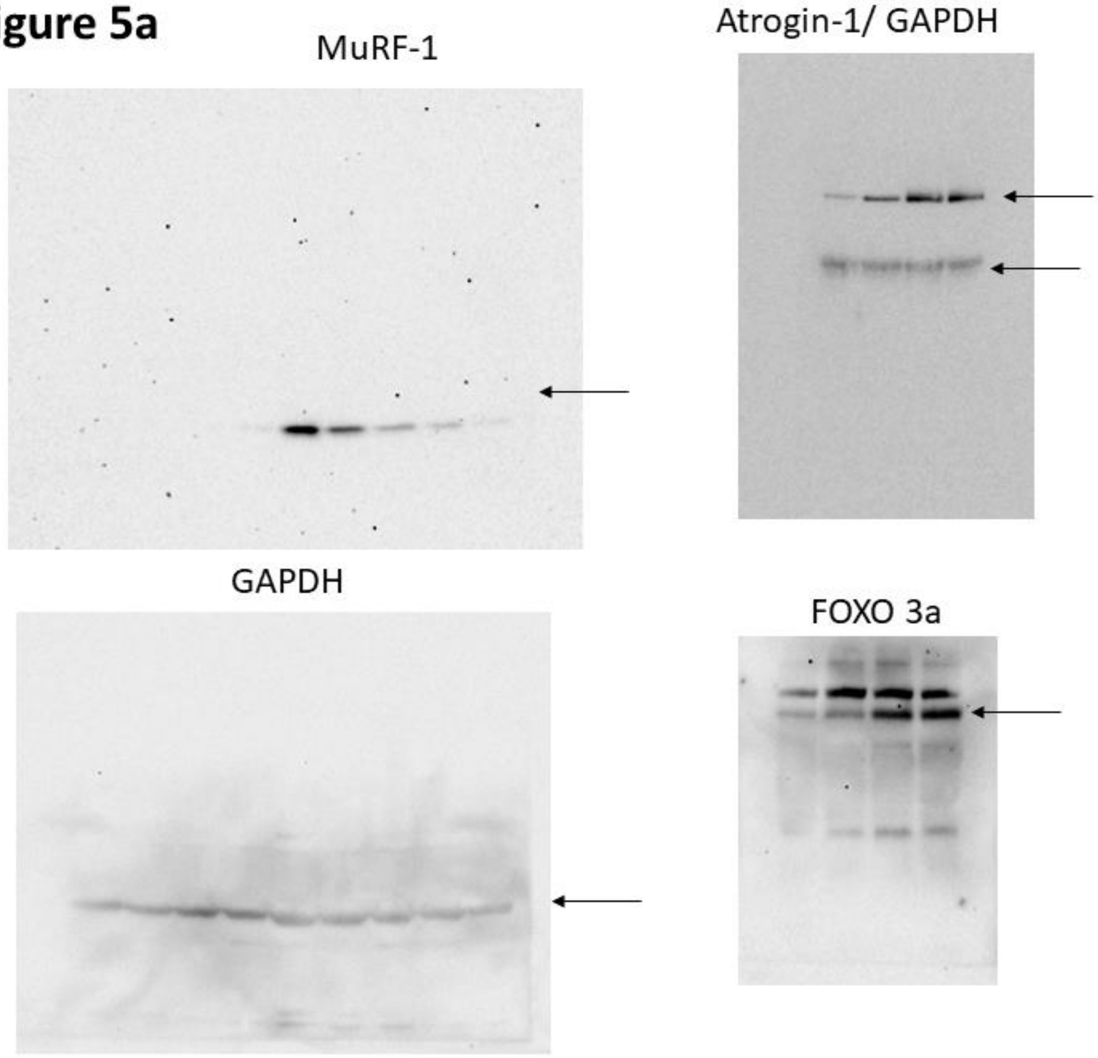

Supplement: Supplementary file 1 — Supplementary Information [file 41598_2018_23669_MOESM1_ESM.pdf]
